# Synergistic effects of grass competition and insect herbivory on the weed *Rumex obtusifolius* in an inundative biocontrol approach

**DOI:** 10.1038/s41598-023-45609-y

**Published:** 2023-10-28

**Authors:** Julie Klötzli, Matthias Suter, Urs Schaffner, Heinz Müller-Schärer, Andreas Lüscher

**Affiliations:** 1 Forage Production and Grassland Systems, Agroscope, 8046 Zurich, Switzerland; 2 Department of Biology/Ecology and Evolution, University of Fribourg, 1700 Fribourg, Switzerland; 3 CABI, 2800 Delémont, Switzerland

**Keywords:** Agroecology, Entomology, Plant ecology

## Abstract

Outcomes of weed biological control projects are highly variable, but a mechanistic understanding of how top-down and bottom-up factors influence the success of weed biological control is often lacking. We grew *Rumex obtusifolius*, the most prominent native weed in European grasslands, in the presence and absence of competition from the grass *Lolium perenne* and subjected it to herbivory through targeted inoculation with root-boring *Pyropteron* spp. To explore whether the interactive effects of competition and inundative biological control were size-dependent, *R. obtusifolius* was planted covering a large range of plant sizes found in managed grasslands. Overall, competition from the grass sward reduced aboveground biomass and final root mass of *R. obtusifolius* about 62- and 7.5-fold, respectively, and increased root decay of *R. obtusifolius* from 14 to 58%. Herbivory alone increased only root decay. However, grass competition significantly enhanced infestation by *Pyropteron* spp. and, as a consequence, enhanced the impact of herbivory on aboveground biomass and final root mass. The synergistic effect was so strong that *R. obtusifolius* plants grown from initially smaller roots did no longer develop. Inoculating *R. obtusifolius* with *Pyropteron* species in grasslands should be further pursued as a promising inundative biological control strategy in the weed’s native range.

## Introduction

In weed management, research has repeatedly focused on understanding and manipulating the relative importance of interspecific plant competition for limiting resources (bottom-up effect) and herbivory (top-down effect)^1–4^. To compare the impact of plant competition and herbivory and analyze their interactive effects, Sheppard proposed three possible outcome categories: substitutive, additive (or multiplicative), and synergistic^5^. While synergistic outcomes, i.e., the impact of the combined application of plant competition and herbivory being higher than the multiplicative effect of the two single factors, are most preferred from a weed control perspective, they are rather uncommon. In most situations, multiplicative outcomes (i.e., additive on the log scale) are observed, with plant competition as the relatively more important factor^5^. Yet, there is a high variability in outcomes of studies combining plant competition and biocontrol herbivory^5,6^, and a mechanistic understanding of how factors interact and how they influence, individually or combined, the success of weed biological control is often lacking. This may explain why the success of such projects varies considerably and ranges from having a negligible impact to resulting in complete control^7^.

**Figure 1:**
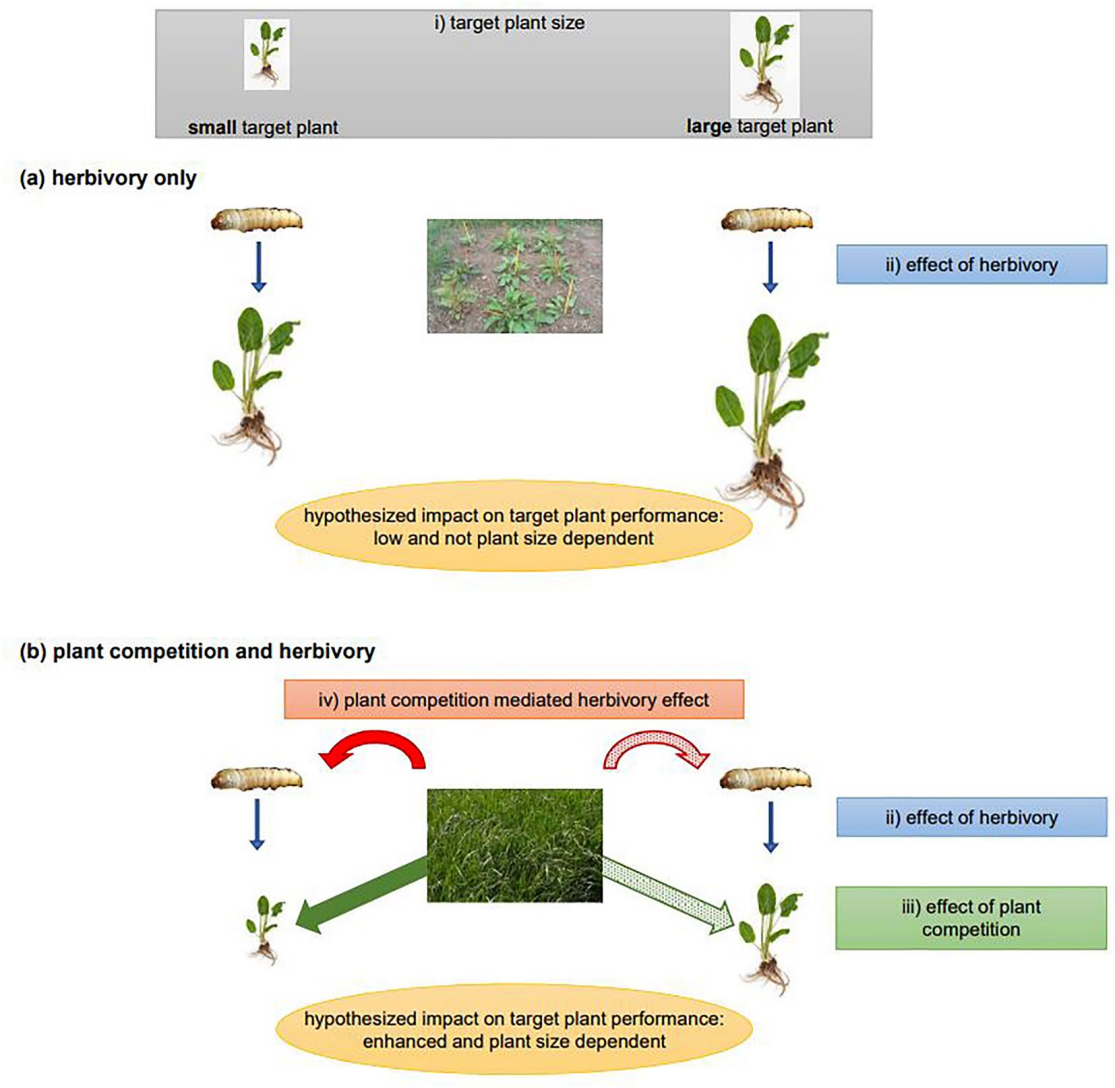
Conceptual illustration of the direct and indirect effects of plant competition and herbivory on a target plant’s performance at two differing initial plant sizes. Hypothesized outcomes under no competition (**a**) and plant competition (**b**) are each displayed in yellow ellipse. In general, herbivory can be expected to impair weed growth across a range of plant sizes (**a**: ii). However, in the absence of interspecific plant competition, weeds may benefit from increased availability of abiotic resources, potentially allowing them to respond to non-lethal herbivory by investing in compensatory or even over-compensatory regrowth^62^. When exposed to the effects of both plant competition and herbivory (**b**: ii, iii), weed performance is expected to be more severely affected than by herbivory alone, mainly because resource availability will be limited. Moreover, effects of plant competition on herbivore behavior and survival may influence the top-down pressure on the target weed (**b**: iv). Where the effects of plant competition and herbivory are plant size dependent (i), the magnitude of the interactive effect of herbivory and plant competition would differ in smaller and larger weed plants (**b**: compare internal structure of arrows).

To advance our understanding of the interactive effects of plant competition and herbivory on weed performance or abundance, not only the direct impacts on the target plant, but also the interactions between competition and herbivory should be studied^6^ (see Fig. 1 for a conceptual illustration). For example, plant competition can mediate either herbivore load (e.g., through a change in plant apparency or micro-climatic conditions^8^) or herbivore impact (e.g., through a change in herbivore defense or tolerance^9^). Alternatively, moderate direct effects of herbivory can lead to significant changes in the competitive interactions between the target weed and the desirable plant community^10,11^. Moreover, herbivore impacts might depend on the size of the target weed, as plant herbivore resistance or tolerance might differ among growth stages. Evaluation of plant size and its inclusion as a covariate into statistical models can thus “reduce the unexplained variance and reveal agent effects that would otherwise have gone undetected”^12^. Yet, to assess plant size dependency of the interactive effects of plant competition and herbivory on weed performance, multi-factorial studies are required that allow investigating direct and indirect processes for a range of pre-specified plant sizes. Although repeatedly demanded (e.g., Morin et al.^12^, Willis et al.^13^) such studies are rare.

*Rumex obtusifolius* L. (broad-leaved dock) is a perennial plant and is considered a problematic weed in both its native range (Eurasia) and its introduced ranges (North and South America, Southern Africa, Australia, New Zealand, Eastern Asia)^14–16^. Several characteristics of the plant facilitate successful propagation, including high production of seeds and their long viability^15^ as well as a taproot system with the ability for clonal growth^17^. Where *R. obtusifolius* is abundant, it can form large, persistent soil seed banks^18^ from which the species can potentially recruit for many years. In conventional farming, *R. obtusifolius* is often controlled by herbicides. However, in organic farming, where herbicides are not allowed, non-chemical control measures are highly demanded^16,19^.

*Rumex obtusifolius* is able to grow in intensively managed grasslands used for livestock production, as the species resists repeated defoliation^16,20^. These grasslands can be sown or permanent and, depending on pedo-climatic conditions, are fertilized and mown and/or grazed several times per year^21,22^. Under medium to high management intensity, weeds are suppressed by resource competition from the forage plants^23–25^. However, although high management intensities with frequent cutting (up to six times annually) reduce aboveground biomass of *R. obtusifolius*, the species cannot be eliminated by cutting alone^16,20,26^. In situations with very high *R. obtusifolius* abundance (> 5–8 small to big plants m^−2^), its negative impact on forage yield becomes so strong that grasslands are generally renewed by ploughing and reseeding^27^. All these aspects point to the need for a cost-effective control of the species.

The two congeneric sesiid moths, *Pyropteron chrysidiforme* (Esper) and* P. doryliforme* (Ochsenheimer) (Lepidoptera; Sesiidae) are native to Europe. *Pyropteron chrysidiforme* occurs throughout large parts of western and central Europe, while *P. doryliforme* is mainly found in the Mediterranean region^28,29^. The larvae of both species are root borers and are highly host-specific to the genus *Rumex*. They were considered as candidates in a classical biological control program in Australia against introduced *Rumex* species, including *R. obtusifolius*^28,29^, but only *P. doryliforme* was able to synchronize its univoltine life cycle to the southern hemisphere conditions. This classical biological control program was successful and resulted in a significant reduction in densities of introduced *Rumex* species^30^. Biological control has also been considered as a non-chemical management option against *R. obtusifolius* in the weed’s native range in Europe by implementing an inundative biological control approach^19,31^. The inundative biological control approach uses periodic releases of large numbers of control agents over a target weed population to control it by causing high levels of damage, while the build-up of antagonist populations is neither intended nor expected^19,32^. However, a recent study in which full-grown *R. obtusifolius* plants were inoculated with eggs of *P. chrysidiforme* under field conditions revealed an insufficient level of control^33^. Root biomass of large *R. obtusifolius* plants was only marginally affected by the herbivore and no mortality was observed^33^.

Given the results of Hahn et al.^33^, here we assessed the interactive effects of interspecific plant competition and root herbivory on *R. obtusifolius* to clarify the extent to which these effects and their relative importance depended on the size of the target weed. To this aim, we manipulated three factors in a field experiment: first, presence or absence of competition from a grass sward (*Lolium perenne* L.); second, root herbivory of larvae by application of the two *Pyropteron* species, *P. chrysidiforme* and *P. doryliforme*; and third, a wide range of root mass (i.e., plant size) of *R. obtusifolius* covering the natural range of size variation. *Lolium perenne* (perennial ryegrass) was chosen as a competitor because it is the most important grass species of temperate productive grasslands worldwide and was previously used to test for competitive effects on *R. obtusifolius*^20,34^. Artificial infestation of *R. obtusifolius* by the two *Pyropteron* species was used to clarify their potential for inundative biological control in the weed’s native range in Europe^19,33^. Finally, target plant size has relevance because large, flowering plants of *R. obtusifolius* are known to be strong competitors^20^, while seedlings or small rosettes are not^35^. Specifically, we addressed the following questions: (1) Does competition from a *L. perenne* sward affect infestation of *R. obtusifolius* by *Pyropteron* larvae (Fig. 1, iv)? (2) What is the relative importance of grass competition and herbivory on the performance of *R. obtusifolius* plants (Fig. 1, ii–iii)? (3) Does grass competition combined with herbivory have a substitutive, multiplicative, or synergistic effect on *R. obtusifolius* performance? (4) Do the interactive effects of competition and herbivory depend on the initial size of *R. obtusifolius* plants (Fig. 1, i)?

## Results

### Increased infestation of herbivores under plant competition

**Figure 2:**
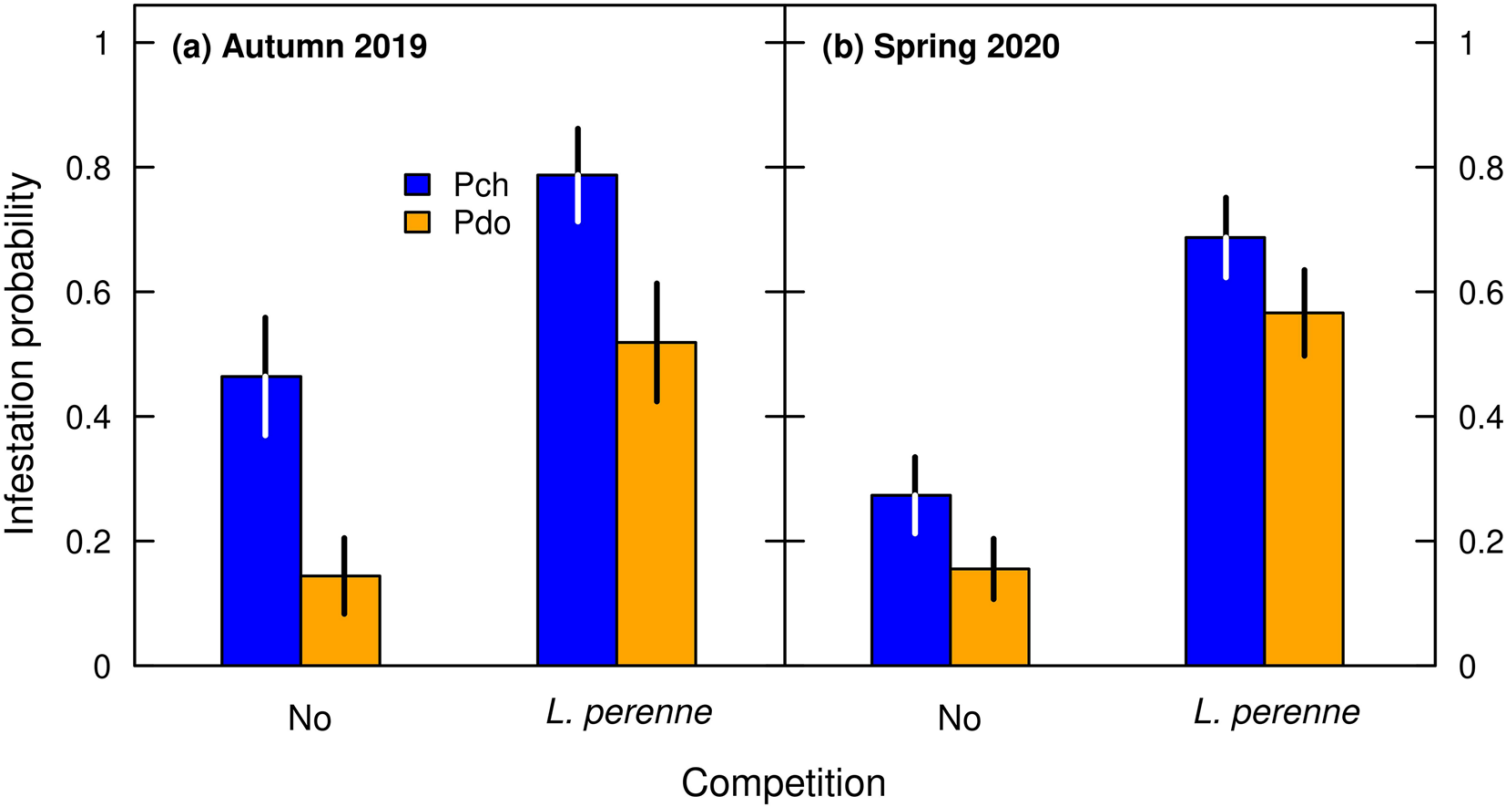
Probability of a *R. obtusifolius* plant being infested by at least one larva measured in the two harvest seasons autumn 2019 (**a**) and spring 2020 (**b**) as affected by *Pyropteron* treatment (application of *P. chrysidiforme* [Pch] or *P. doryliforme* [Pdo]) under no competition and competition from a *L. perenne* sward. Only plants under the two *Pyropteron* treatments analyzed because there was no infestation of control plants (no inoculation with *Pyropteron*). Displayed are means ± standard error across all initial root sizes, calculated following Agresti and Coull^63^.

**Figure 3:**
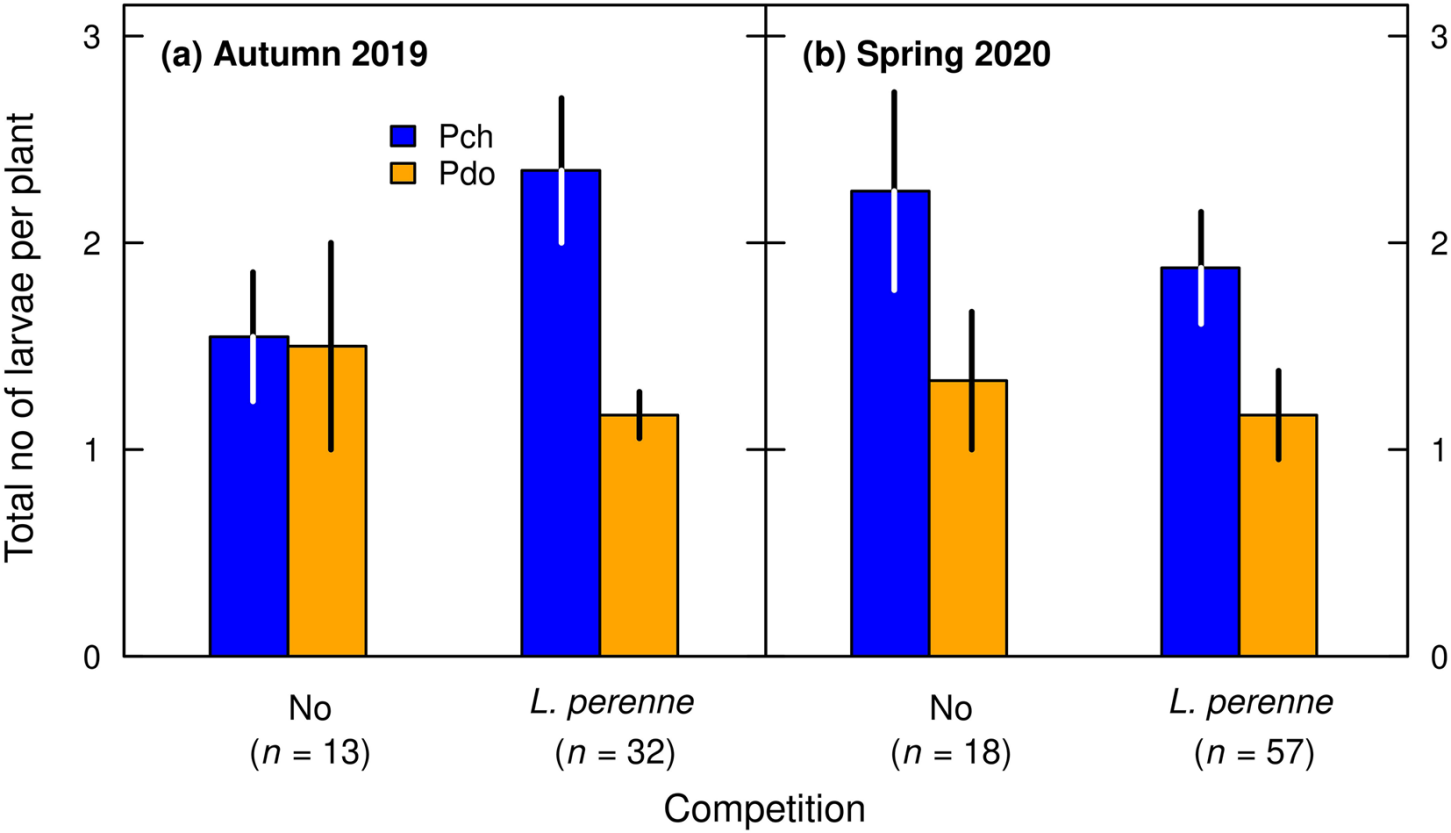
Total number of larvae retrieved per infested plant of *R. obtusifolius* measured in the two harvest seasons autumn 2019 (**a**) and spring 2020 (**b**) as affected by *Pyropteron* treatment (application of *P. chrysidiforme* [Pch] or *P. doryliforme* [Pdo]) under no competition and competition from a *L. perenne* sward. Only infested plants included in analysis (compare *n* below panels). Displayed are means ± standard error across all initial root sizes.

First, we assessed the probability of a *R. obtusifolius* plant being infested by *Pyropteron* after placing a toothpick with 30 eggs glued-on into the soil near the plant base. Infestation probability was significantly greater when *R. obtusifolius* was grown under competition from *L. perenne* (Fig. 2, Table 1: competition effect). For example, in spring 2020, the infestation probabilities were 0.70 and 0.57 for *P. chrysidiforme* and *P. doryliforme*, respectively, when grown under competition, but were 0.26 and 0.13 when grown alone. The degree of infestation was generally sustained over both harvest seasons (Table 1), but infestation was significantly greater for *P. chrysidiforme* than for *P. doryliforme*, with the differences becoming less pronounced in spring 2020 (Fig. 2, Table 1: harvest season × *Pyropteron* interaction). Noteworthy, the infestation probability did not differ between *R. obtusifolius* plants grown from initially smaller or larger roots (Table 1). Roots in the control treatment revealed no signs of herbivore attack, indicating that there was no occasional infestation by *Pyropteron* or other root-boring insects. Second, the total number of larvae retrieved per infested plant was similar under the two competition treatments and between harvest seasons, but was significantly greater for *P. chrysidiforme* than for *P. doryliforme* (Fig. 3, Table 1).

**Table 1:** Summary of generalized linear mixed-effects models for the effects of harvest season, competition from a *L. perenne* sward, *Pyropteron* application treatment, and initial root mass of *R. obtusifolius* on the probability of a *R. obtusifolius* plant being infested and the total number of larvae per infested plant.

Variable	Infestation^#^			Total number of larvae^§^		
	df	χ^2^	*P*	df	χ^2^	*P*
Log(Initial root mass)	1	2.1	0.144	1	10.1	0.001
Harvest season	1	1.9	0.166	1	< 0.1	0.826
Competition	1	32.9	< 0.001	1	0.1	0.761
*Pyropteron* treatment	1	13.3	< 0.001	1	8.6	0.003
Harvest season × Competition	1	< 0.1	0.914	1	2.2	0.139
Harvest season × *Pyropteron* treatment	1	3.6	0.056	1	< 0.1	0.843
Competition × *Pyropteron* treatment	1	0.4	0.516	1	0.2	0.695
*R*^2^_m_		0.336			0.198	
*R*^2^_c_		0.455			0.213	

*R*^2^_m_ and *R*^2^_c_: marginal and conditional *R*^2^, respectively, following Nakagawa and Schielzeth^57^ and Nakagawa et al.^58^. *R*^2^_m_: variance explained by fixed effects; *R*^2^_c_: variance explained by fixed and random effects.

^#^Only plants under the two *Pyropteron* application treatments analyzed because there was no infestation of control plants (no inoculation with *Pyropteron*); df related to the *Pyropteron* treatment adjusted accordingly.

^§^Only infested plants analyzed; df related to the *Pyropteron* treatment adjusted accordingly.

### Synergistic interactions between plant competition and herbivores increase impact on small *R. obtusifolius* plants

**Figure 4:**
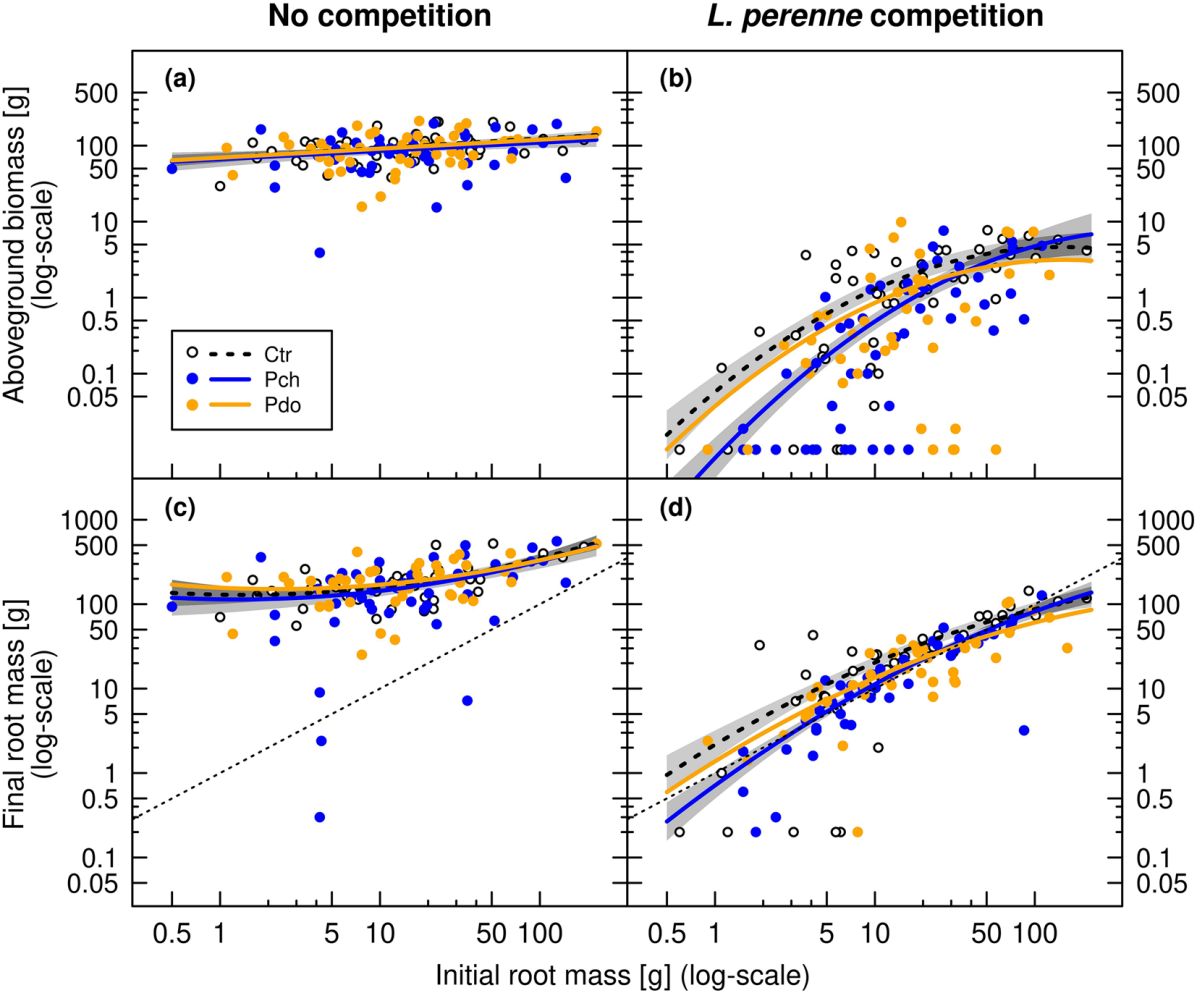
Aboveground biomass and final root mass of *R. obtusifolius* plants in spring 2020 grown under no competition (**a**,**c**) and competition from a *L. perenne* sward (**b**,**d**) depending on the initial root mass and *Pyropteron* treatment (no application [Ctr], application of *P. chrysidiforme* [Pch] or *P. doryliforme* [Pdo]). Predicted lines (± standard error grey shaded) are based on generalized linear mixed-effects models (Table 2). No standard error is given for Pdo because it was largely overlapping with the other treatments. Unequal variances were modeled with a distinct dispersion function (see Supplementary Appendix S1 for the equation). Aboveground biomass is the sum over all five harvests. Dotted lines in panels (**c**) and (**d**) represent the 1:1 relation of final versus initial root mass, i.e., steady-state of growth and decay.

Given that infestation probability (Fig. 2) and larval load (Fig. 3) were similar in autumn 2019 and spring 2020, all further analyses were performed using the data from the final harvest in spring 2020. Here, competition from the *L. perenne* sward reduced the aboveground biomass of *R. obtusifolius* 62.7-fold (Fig. 4a,b) and final root mass 7.4-fold (Fig. 4c,d; Table 2). Thus, on average across the *Pyropteron* treatments, aboveground biomass and final root mass of *R. obtusifolius* were 94.1 g (± 3.74 g SE) and 190.7 g per plant (± 9.80 g), respectively, when grown without competition, but only 1.5 g (± 0.17 g) and 25.7 g (± 2.33 g) when grown under *L. perenne* competition (Fig. 4). The competition effect from *L. perenne* was so strong that there was hardly any growth of *R. obtusifolius* roots under this treatment, irrespective of initial root mass (Fig. 4d: compare dotted line). By contrast, when grown without competition, *R. obtusifolius* plants from all initial root sizes grew up to the same size order of magnitude (Fig. 4a,c).

**Table 2:** Summary of generalized linear mixed-effects models for the effects of competition from a *L. perenne* sward, *Pyropteron* application treatment, and initial root mass of *R. obtusifolius* on its aboveground biomass and final root mass in spring 2020.

Variable	df	Aboveground biomass^#^		Final root mass	
		χ^2^	*P*	χ^2^	*P*
Log(Initial root mass), (linear)	1	21.6	< 0.001	53.8	< 0.001
Log(Initial root mass)^2^, (quadratic)	1	0.1	0.747	3.2	0.071
Competition	1	175.0	< 0.001	131.8	< 0.001
*Pyropteron* treatment	2	2.8	0.253	4.9	0.087
Competition × *Pyropteron* treatment	2	6.6	0.037	6.1	0.048
Log(Initial root mass) × Competition	1	38.0	< 0.001	44.0	< 0.001
Log(Initial root mass)^2^ × Competition	1	7.1	0.008	6.1	0.014
*R*^2^_m_		0.928		0.845	
*R*^2^_c_		0.932		0.858	

*R*^2^_m_ and *R*^2^_c_: marginal and conditional *R*^2^.

^#^Cumulative biomass over five harvests.

*Pyropteron* application negatively affected aboveground biomass and root mass of *R. obtusifolius* when grown under *L. perenne* competition, but not without (Fig. 4, Table 2: significant competition × *Pyropteron* interaction), indicating a synergistic effect of grass competition and *Pyropteron* application on the growth of *R. obtusifolius* plants. The different *Pyropteron* effects in the presence and absence of competition justified to split the data into the ‘*L. perenne* competition’ and ‘no competition’ treatments to reduce model complexity and allow for a clearer interpretation (see Supplementary Appendix S1 for details). By doing so, it turned out that only under competition were plants with initially smaller roots more negatively affected by *Pyropteron* than plants with initially larger roots (Supplementary Table S1: initial root mass × *Pyropteron* interaction), and that the impact was only significant for *P. chrysidiforme* (Fig. 4b,d; Supplementary Table S2). Thus, under *L. perenne* competition, comparing the responses over initial root mass between the control treatment and plants under *P. chrysidiforme* application (reflected in the fitted lines, Fig. 4b,d), a significant slope difference appeared for aboveground biomass (z = 2.3, *P* = 0.022) and final root mass (z = 1.9, *P* = 0.057; Supplementary Table S2), indicating a greater herbivore impact when *R. obtusifolius* was grown from smaller plants. With *P. doryliforme* application, no differences in slopes to the control were evident for aboveground biomass and final root mass (both z ≤ 0.155, *P* > 0.877).

### Competition-mediated herbivore load per final root mass

**Figure 5:**
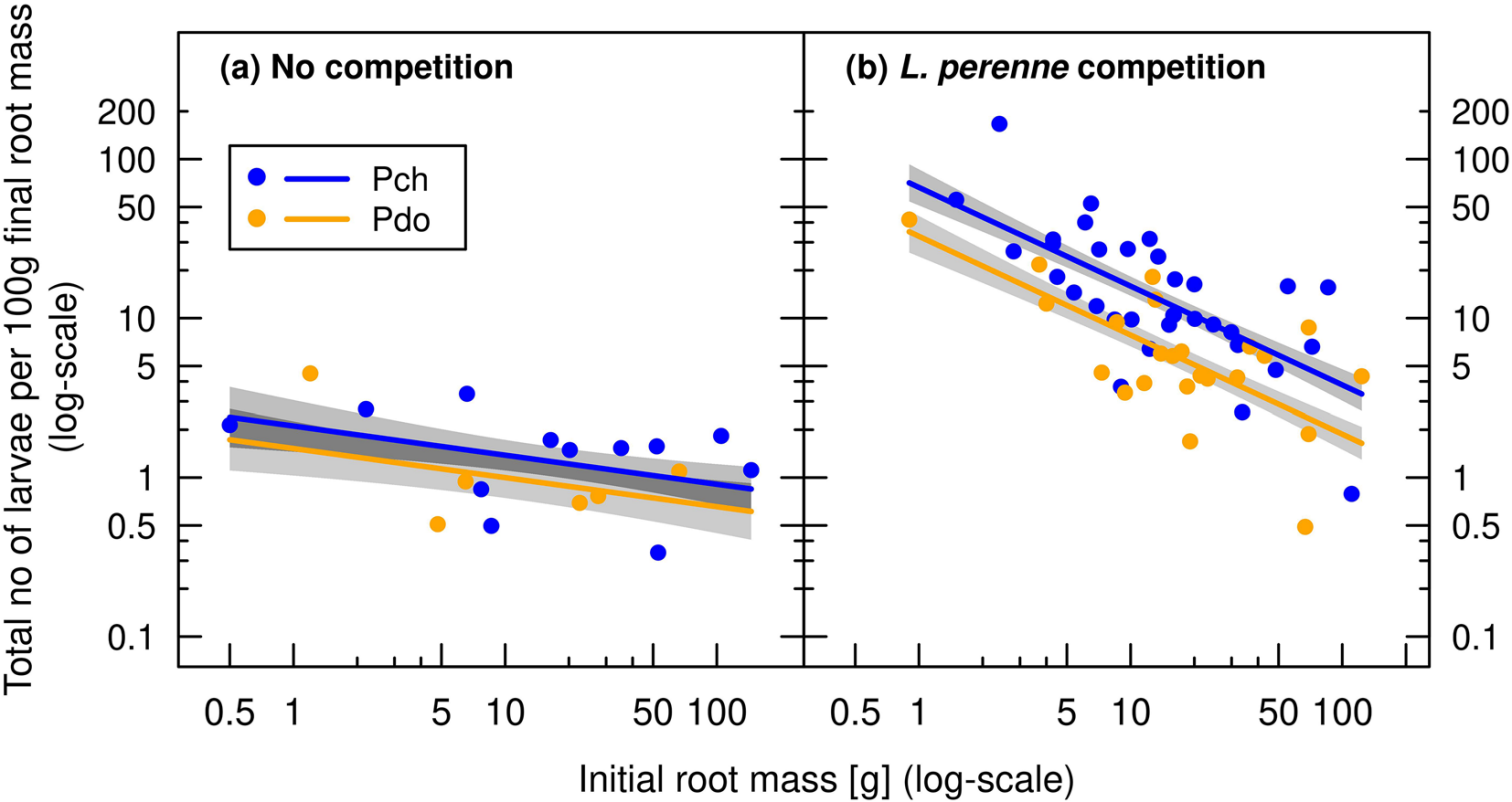
Number of larvae retrieved per 100 g final root mass of infested *R. obtusifolius* plants in spring 2020 grown under no competition (**a**) and competition from a *L. perenne* sward (**b**) depending on the initial root mass and *Pyropteron* treatment (application of *P. chrysidiforme* [Pch] or *P. doryliforme* [Pdo]). Only infested plants included in analysis. Predicted lines (± standard error grey shaded) are based on a linear mixed-effects model (Table 3).

Combining the results of number of larvae per plant (Fig. 3) and the effects of competition and *Pyropteron* application on root mass (Fig. 4c,d) suggested that herbivore loads per unit root mass would differ between small and large roots at the end of the experiment. Indeed, when the number of larvae retrieved was scaled by 100 g of final root mass, significant differences between the competition treatments were evident (Fig. 5, Table 3: competition effect). In spring 2020, on average 1.6 and 1.3 larvae per 100 g final root mass were retrieved for *P. chrysidiforme* and *P. doryliforme*, respectively, under no competition, but 15.5 and 7.4 larvae (per 100 g final root mass) under *L. perenne* competition. Thus, competition from the grass sward induced a ten- and sixfold higher herbivore load per unit final root mass for *P. chrysidiforme* and *P. doryliforme*, respectively, the difference in larval load between the two *Pyropteron* species being significant (Table 3). Furthermore, significantly more larvae per unit final root mass were retrieved from initially smaller roots than from larger roots when plants were grown under competition from *L. perenne* (Fig. 5b, Table 3: initial root mass × competition interaction).

**Table 3:** Summary of linear mixed-effects model for the effects of competition from a *L. perenne* sward, *Pyropteron* application treatment, and initial root mass of *R. obtusifolius* on the number of larvae scaled by final root mass of infested *R. obtusifolius* plants in spring 2020.

Variable	No of larvae per 100 g final root mass^#^			
	df_term_	df_res_	F	*P*
Log(Initial root mass)	1	67.9	38.6	< 0.001
Competition	1	69.8	104.3	< 0.001
*Pyropteron* treatment	1	65.2	15.3	< 0.001
Competition × *Pyropteron* treatment	1	65.2	0.9	0.341
Log(Initial root mass) × Competition	1	63.1	9.9	0.003
*R*^2^_m_			0.724	
*R*^2^_c_			0.743	

df_term_: degrees of freedom of term; df_res_: degrees of freedom of residuals; *R*^2^_m_ and *R*^2^_c_: marginal and conditional *R*^2^.

^#^Only infested plants analyzed; df related to the *Pyropteron* treatment adjusted accordingly.

### Synergistic interactions increase root decay

**Figure 6:**
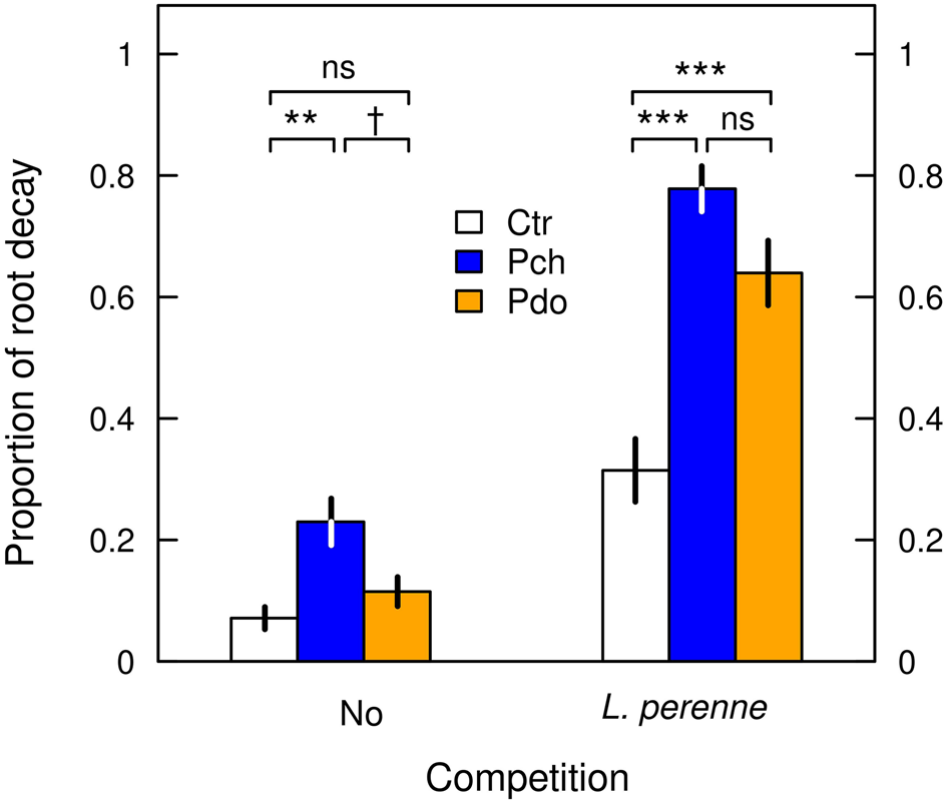
Proportion of root decay of *R. obtusifolius* in spring 2020 as affected by *Pyropteron* treatment (no application [Ctr], application of *P. chrysidiforme* [Pch] or *P. doryliforme* [Pdo]) under no competition and competition from a *L. perenne* sward. Displayed are means ± standard error across all initial root sizes. Symbols indicate significance of Tukey range test within each competition treatment, following a generalized linear mixed-effects model (Table 4). ***P ≤ 0.001, **P ≤ 0.01, ^†^*P* ≤ 0.1, *ns* not significant.

The proportion of root decay was significantly higher in *R. obtusifolius* plants grown under *L. perenne* competition than in those grown without competition, and it was significantly increased by *Pyropteron* application (Fig. 6, Table 4). Furthermore, competition from *L. perenne* amplified root decay caused by *Pyropteron* application (Table 4: marginally significant competition × *Pyropteron* interaction), again pointing to a synergistic effect between the two factors. The average proportions of root decay under no competition were 0.07, 0.23, and 0.11 for control, *P. chrysidiforme*, and *P. doryliforme* treatments, respectively, but under competition from *L. perenne* were, respectively, 0.31, 0.78, and 0.64. Initially smaller roots revealed significantly greater root decay than initially larger roots only under the competition treatment (Table 4: initial root mass × competition interaction, no figure shown).

**Table 4:** Summary of a generalized linear mixed-effects model for the effects of competition from a *L. perenne* sward, *Pyropteron* application treatment, and initial root mass of *R. obtusifolius* on its proportion of root decay in spring 2020.

Variable	Proportion root decay		
	df	χ^2^	*P*
Log(Initial root mass)	1	1.4	0.229
Competition	1	64.2	< 0.001
*Pyropteron* treatment	2	34.1	< 0.001
Competition × *Pyropteron* treatment	2	4.8	0.092
Log(Initial root mass) × Competition	1	6.9	0.009
*R*^2^_m_		0.392^#^	
*R*^2^_c_		0.393^#^	

*R*^2^_m_ and *R*^2^_c_: marginal and conditional *R*^2^.

^#^Approximation by squared correlation of linear predictor and link-transformed response.

**Figure 7:**
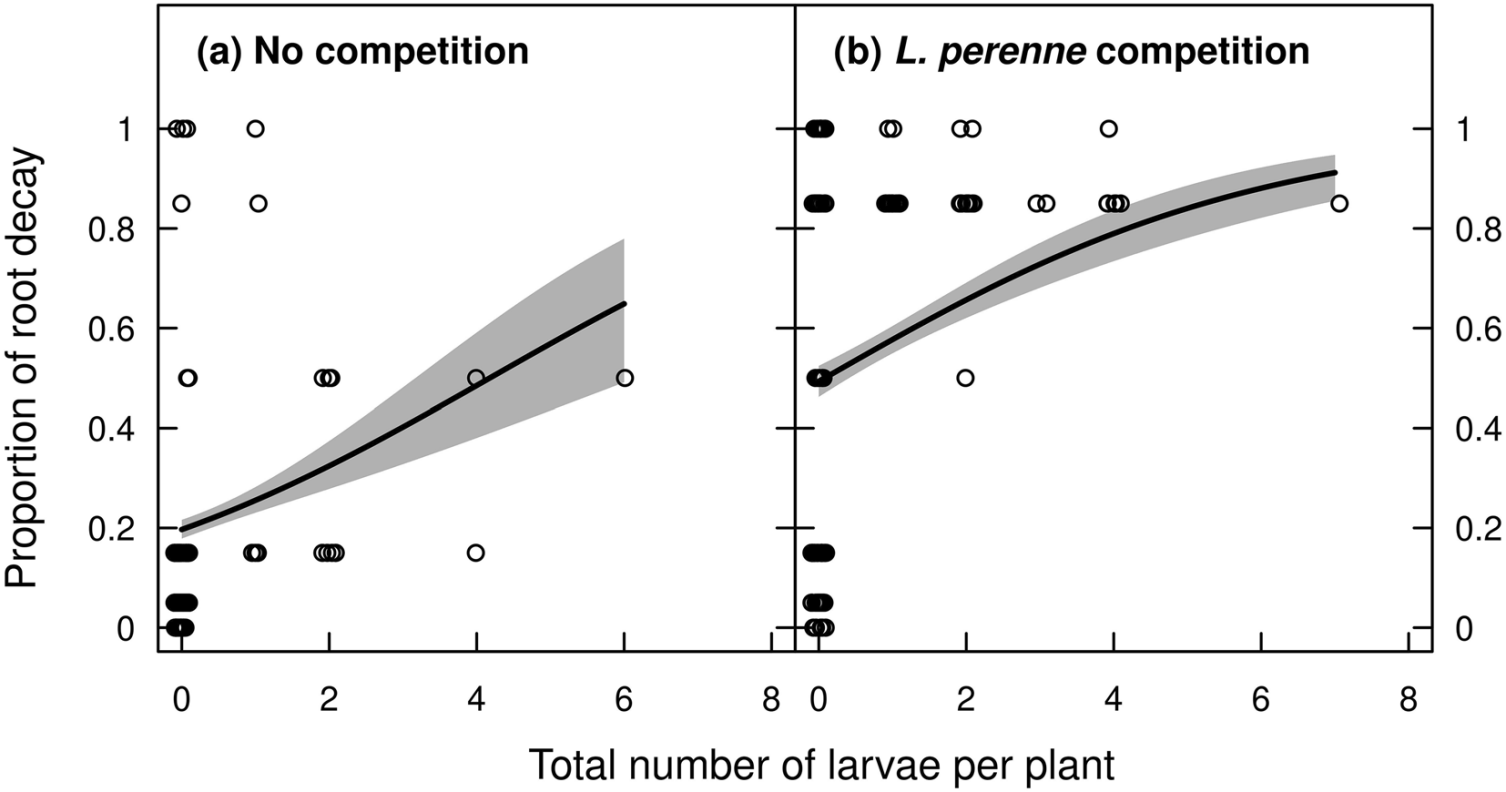
Proportion of root decay of *R. obtusifolius* in spring 2020 under no competition (**a**) and competition from a *L. perenne* sward (**b**) depending on the total number of larvae per infested plant. Predicted lines (± standard error grey shaded) are from a beta regression model with an overall *R*^2^ value of 0.31. Fitted slopes were not significantly different between competition treatments (χ^2^ < 0.1, df = 1, *P* = 0.995). Circles are scattered horizontally to improve their visibility.

The proportion of root decay was positively related to the number of larvae retrieved under both competition treatments (*z* = 4.9, *P* < 0.001, inference for slopes in Fig. 7). However, per unit number of larvae, greater proportional root decay was caused in plants grown under *L. perenne* competition than those grown without competition (*z* = 8.7, *P* < 0.001, inference for average level of root decay, Fig. 7).

### No compensatory growth of *R. obtusifolius* plants due to herbivory

Competition from *L. perenne* significantly suppressed the formation of auxiliary rosettes of *R. obtusifolius* (Supplementary Fig. S1 and Table S3) and reduced the number of roots (Supplementary Fig. S2 and Table S3), while *Pyropteron* application caused no effects on either parameter. Thus, despite the impact of the *Pyropteron* larvae on growth of aboveground biomass and root mass of *R. obtusifolius* (Fig. 4) and proportion of root decay (Fig. 6), *R. obtusifolius* plants did not respond with compensatory growth.

## Discussion

Using a multi-factorial experimental design, we found that interspecific competition and targeted application of biological control candidates affected the growth of *R. obtusifolius* in an interactive, synergistic way. Plant competition had a higher direct impact on *R. obtusifolius* growth than herbivory and also indirectly affected plant growth by increasing the probability of infestation by the biological control candidates and, consequently, the level of root decay. By integrating the range of naturally occurring plant sizes, we could show that an additional amount of the observed variation in interactive effects of competition and herbivory can be explained by plant size. Smaller plants were affected by both competition and herbivory by *P. chrysidiforme*, while larger plants were mainly affected by competition. These results provide evidence for a significant plant size dependency in the magnitude of the effects of competition and herbivory on the growth of *R. obtusifolius* and have implications for the management of this problematic weed.

Multi-factorial approaches enable identification of the relative effects of several factors in a single ecological context and whether substitutive, multiplicative or synergistic dynamics are associated with the combination of factors. In our study, interspecific plant competition was the single most important factor impairing growth of *R. obtusifolius* plants, which is consistent with previous studies comparing the effect of competition and herbivory on weed performance^5,36,37^. *Lolium perenne*, a characteristic grass species of productive grassland systems in temperate regions worldwide, was able to effectively suppress growth of *R. obtusifolius* and to increase root decay. In a similar study assessing the combined effects of grass competition and root herbivory on *Centaurea maculosa* Lam.^38^, the strong effect of the grass competitor was attributed to belowground competition for nutrients and water, as well as competition for light of the rosette plants in the grass sward. In our experiment, we specifically tested interactive effects of interspecific plant competition and herbivory at two fixed levels (i.e., presence vs. absence). Theoretically, the effects of both plant competition and herbivory may vary with plant density/biomass or herbivore load. In the case of interspecific plant competition, however, applying different sowing densities of *L. perenne* to intensively managed grasslands (as reflected in our experiment) would not translate into differences in yield or resource use^21,39^. Therefore, manipulating sowing density of *L. perenne* cannot be expected to result in differential competitive effects on *R. obtusifolius*^34^.

Herbivory affected the performance of *R. obtusifolius* to a lesser extent than plant competition. The clearest effect of herbivory was on proportion of root decay, which increased through feeding by *P. chrysidiforme* larvae in both the presence and absence of plant competition (Figs. 6 and 7). Root decay is expected to translate into a reduction in plant performance^40^ and in our study, increase in root decay coincided with reduced belowground and aboveground biomass production (compare Figs. 4 and 6). Despite the percentage of root decay being high, *R. obtusifolius* plants did not respond with compensatory growth to *Pyropteron* feeding (Supplementary Table S3, Figs. S1, S2). Thus, a plant with 70% decayed root mass (approx. mean of *Pyropteron* applications under competition, Fig. 6) can be assumed to be severely degraded if not dead, which should result in a sustained herbivory effect in the long run. It should be noted that in our study the effect of herbivory on root biomass was underestimated, as the root biomass measured at the end of the experiment also included degraded root mass. Infestation probability and impact were higher with *P. chrysidiforme* than with *P. doryliforme*, which may indicate differences in climatic suitability of the two species. *P. chrysidiforme* has its distribution range in central and western Europe and is better adapted to the relatively cool and humid conditions of the study site than *P. doryliforme,* which has a Mediterranean distribution range^29^. Finally, the number of larvae per plant (Fig. 3) was in the same range as found in previous studies^33,41^, indicating that *P. chrysidiforme* and *P. doryliforme* build up comparable herbivore loads under varying field conditions.

Under natural conditions, plant competition and other factors may also affect oviposition behavior and thus increase variation in number of eggs deposited on *R. obtusifolius* plants. Here, we deliberately applied a standardized number of eggs to reflect a biocontrol management approach using targeted inoculations of individual plants and by this, aspects of host-finding and female oviposition preferences depending on host size and occurrence of non-host species have been excluded. The number of eggs applied were in the range of those used in the biological control program in Australia^42^ and in a previous inundative biological control experiment in Switzerland^33^. Moreover, preliminary tests revealed that applying a different number of eggs per plants did not result in significantly different infestation rates (U. Schaffner, unpublished data). This may be partly explained by interference competition among larvae as observed in laboratory bioassays and pot experiments^43^.

In addition to the direct effects of plant competition, we found that plant competition also indirectly affected growth of *R. obtusifolius* by increasing the probability of infestation by *Pyropteron* larvae (Fig. 2, up to 70% infested plants in spring 2020), indicating that plants suppressed by competition experienced a stronger herbivore effect. Theoretically, neighboring plants may negatively (associational resistance) or positively (associational susceptibility) interact by altering the behavior or numerical responses of their natural enemies^1,44,45^. Shabbir et al. found that a biological control agent for the weed *Parthenium hysterophorus* L. induced more galls when the target weed was grown in competition with other plant species than when grown alone^46^; the authors proposed that the increased herbivore load on plants under competition may be due to a fertilizer effect of the competing plants or due to altered female oviposition behavior. In our study, altered female oviposition behavior could not explain the increased herbivore load and root decay, as all plants subjected to herbivory were inoculated with the same number of eggs. The grass sward surrounding the inoculated plants may, however, have improved the micro-climatic conditions for neonate larvae thus increasing their survival from egg hatching to entering the roots, resulting in higher infestation (Fig. 2). In an experimental study, herbivore damage on *Solanum carolinense* increased with increasing frequency of a plant competitor, and soil moisture and other microclimate variables were (negatively) correlated with herbivore damage^47^, suggesting that the susceptibility was at least partly mediated by effects of plant competition on microclimatic conditions. An alternative explanation for associational susceptibility is that plant competition and the associated reduction in resource availability may impair the target plant’s investment in chemical defenses^48^. In our study, the increased infestation rate of *R. obtusifolius* by the herbivores under *L. perenne* competition constitutes a mechanism that partially explains the synergism between the two suppressive factors.

Following the classification of Sheppard on the type of outcomes^5^, the combination of plant competition and root herbivory enhanced the negative effect on *R. obtusifolius* in a synergistic way (Figs. 4 and 6, Tables 2 and 4). Synergism between the two factors can also be demonstrated by calculating the effect size based on the single, isolated effects (i.e., the effect of competition without *P. chrysidiforme* application and of *P. chrysidiforme* application in the absence of competition) and comparing this value with the observed impact under the combined treatment. Doing so for aboveground biomass grown from the two pooled largest categories of initial root mass, we expected a 29-fold reduction from the multiplied isolated effects, but observed a 50-fold reduction with the combined treatment. The same figure for aboveground biomass grown from the two pooled smallest initial root categories predicted a 196-fold reduction from the multiplied isolated effects, but we observed a 1067-fold reduction with the combined treatment. Our findings are consistent with the simpler type of synergy where the factor with the lower interaction strength (usually herbivory) is observed to have an impact only in the presence of the factor with the higher interaction strength (usually plant competition^4,5,40^). In our experiment, the number of larvae per plant was not affected by the competition treatment (Fig. 3, Table 1). However, once established in the root, these larvae had a generally higher impact on *R. obtusifolius* plants suppressed by the *L. perenne* sward (Fig. 4), which can be explained by the higher larval load per unit final root mass (Fig. 5). Surprisingly, field data for the existence of synergistic effects of herbivory and plant competition are relatively rare and, even if demonstrated, it is not clear whether these synergistic effects are strong enough to effectively control native weeds or invasive alien plant species (but see Santamaría et al.^49^, Zhang et al.^50^).

By deliberately incorporating a gradient of a trait of the target weed, i.e., initial plant size, we have shown that the interactive effects of competition and herbivory are subject to non-linear dynamics (Fig. 4). We chose initial plant size as a variable because it reflects a major source of variation in plant communities and because literature underlines the role of plant size in mediating plant competition^20,51^ and herbivory^4^. In *R. obtusifolius*, root size relates to carbohydrate storage capacity and therefore to the potential to resist sustained stress^17,52^, and in our study, larger plants appeared to have, at least in the short-term, a higher tolerance to herbivory even when subjected to competition. The observed effects imply that interspecific competition for soil resources and light, in combination with root herbivory, exhausted small plants to the degree that root growth was no longer possible (Fig. 4d) and plants would eventually die (J. Klötzli, personal observation). Our results also demonstrate that incorporating species traits and other biotic or abiotic variables can help increase the predictability of the interactive effects of herbivory and competition on plant performance. Plant size dependency has rarely been systematically investigated using multifactorial experiments that evaluate different biotic and abiotic factors on the joint effects of herbivory and (interspecific) plant competition. In our model, initial plant size explained an additional amount of variation in the interactive effects of competition and herbivory on *R. obtusifolius* growth, and by ignoring incorporation of initial plant size into the model, erroneous conclusions could have been drawn, thereby affecting management recommendations.

### Implications for management

Regarding practical management, combining competition from grass swards and herbivory to manage grassland weeds in general and particularly *R. obtusifolius* has received interest in the past^34,53^ and follows the strategies of integrated weed management^37,54^. In the case of perennial weeds, herbivores attacking the storage organs belowground represents a great option to successfully reduce weed performance^55^. In our study, for example, the percent reduction of final aboveground biomass and root mass of *R. obtusifolius* induced by *P. chrysidiforme* was -85% and -80%, respectively, for the two pooled smallest categories of initial root mass. Such a strong impact suggests that *P. chrysidiforme* inoculated using an inundative approach in an established grassland could be a promising control option. This approach is also compatible with organic agriculture, where measures for the control of *R. obtusifolius* are urgently needed^19^. However, *P. chrysidiforme* would need to be repeatedly applied because a single application will most probably not permanently change the herbivore density to a degree as to result in an enduring impact on *R. obtusifolius* population densities. Rather, the results of this study suggest that the combination of interspecific competition and herbivory primarily damage smaller *R. obtusifolius* plants, which in the short-term would lead to a change in the size distribution of *R. obtusifolius* populations. The control effect will arise over time, when establishment of younger plants is impeded by repeatedly applied herbivores and mature plants naturally die or are regulated by other means. Our study thus indicates that smaller plants should be targeted using this inundative approach. It remains to be shown whether several years of competition and herbivory will further increase root decay, thus also weakening large *R. obtusifolius* plants.

## Methods

### Study system

Eggs of the two *Pyropteron* species used in this study originated from rearing colonies maintained at the CABI Switzerland Centre, Delémont. The rearing colony of *P. chrysidiforme* was established in 2010 with insects collected in southwestern Switzerland, while the colony of *P. doryliforme* was established in 2018 with insects collected in southern Spain. *Rumex obtusifolius* plants used in the experiment were dug up from managed, productive grasslands in northern Switzerland, where neither of the two *Pyropteron* species occur naturally. The use of *Pyropteron* spp. and *R. obtusifolius* plants in the present study complies with international, national and institutional guidelines, and we had the consent from the farmers who owned and managed the grasslands to sample *R. obtusifolius* plants.

### Site conditions and experimental layout

A field experiment was carried out at Agroscope Zürich-Reckenholz, in central Switzerland (47°43′79″ N, 8°52′81″ E, 486 m a.s.l). The soil at the site is classified as *calcic cambisol* with a topsoil composition of 37.6% sand, 39.2% silt, 21.0% clay, containing 2.2% humus, and with a pH of 6.6. Experimental plots were established in spring 2019 and were maintained until May 2020. In 2019, mean annual temperature was 10.6 °C and annual precipitation 972 mm.

The experimental layout followed a split-split plot design with three factors to test the effects of plant competition, application of herbivore species, and initial root size of *R. obtusifolius* on its performance. Swards of *Lolium perenne* L. cultivar Allodia and plots with bare soil were established on sixteen main-plots of 1.8 m × 5 m (main-level factor), termed ‘*L. perenne* competition’ and ‘no competition’ treatment, respectively (see below for details on the treatments). Within each main-plot, three *Pyropteron* treatments testing the herbivore species were randomly assigned to sub-plots of 1.8 m × 1.66 m: application of *P. chrysidiforme* to *R. obtusifolius* plants, application of *P. doryliforme*, and a control with no application of either herbivore (split-level factor). Within each sub-plot, nine roots of *R. obtusifolius* of different size were randomly assigned to a 3 × 3 grid with a spacing of 0.4 m (split-split level factor), thus preventing plant-to-plant movement of herbivores and assuring that *L. perenne* remained the most abundant plant component in the competition treatment. This resulted in a total of 432 planted roots. Main-plots were arranged according to a randomized complete block design on the site (8 blocks, each containing a *L. perenne* competition and a no competition treatment).

### Treatments and management

In the competition treatment, swards of *L. perenne* were sown after soil tilling in mid-August 2018 at a density of 25 kg seeds ha^−1^. Plots with bare soil representing the no competition treatment were tilled, but not seeded and were regularly weeded thereafter to prevent the growth of plants other than *R. obtusifolius*.

In early February 2019, *R. obtusifolius* plants of different sizes were dug up from grasslands (locations as noted), and roots were carefully washed free of soil and stored in a dark, cold room at 4 °C until early June. Thereafter, all roots were weighed, cut to a maximum length 15 cm, and labeled. The mass of these roots followed a log normal distribution, and the total sample was split into nine approximately equal groups based on log root mass. In mid-June 2019, roots were planted into the soil close to the surface: one root randomly selected from each size category was (randomly) arranged into the 3 × 3 grid of a sub-plot, for a total of nine plants per grid.

In early July 2019, after a three weeks period during which the roots were allowed to settle to the soil, all *R. obtusifolius* plants in the respective treatments were infested with *P. chrysidiforme* or *P. doryliforme* by placing a toothpick with 30 eggs glued-on into the soil near the plant base, following a procedure successfully implemented by Fisher et al.^42^. The toothpicks were removed after 2 weeks. The mean hatching rate per toothpick, determined by evaluating the eggs, was 0.85 and 0.75 for *P. chrysidiforme* and *P. doryliforme*, respectively, with no difference between the competition treatments.

All plots received phosphorus (100 kg P ha^−1^ year^−1^) and potassium fertilizer (100 kg K ha^−1^ year^−1^) in early spring 2019, following local fertilization recommendations for intensively managed grasslands. Moreover, plots were fertilized at a rate of 150 kg nitrogen ha^−1^ year^−1^ over three equal applications in mid-August 2019, mid-September 2019, and mid-March 2020. Applications in August and September were 3 to 5 days after cutting aboveground biomass of *R. obtusifolius* plants and mowing *L. perenne* swards to a height of 7 cm with a plot harvester (Wintersteiger Cibus).

### Measurements

Aboveground biomass of all *R. obtusifolius* plants was repeatedly cut at a height of 5 cm aboveground, when the largest plants reached the flowering stage: in August, September, and November 2019, as well as in April and May 2020. The harvested biomass was dried at 105 °C for 24 h and weighed. For each plant, aboveground biomass was summed over harvests to obtain the cumulative aboveground dry biomass.

To evaluate the immediate infestation success of the herbivore species, one third of the *R. obtusifolius* roots were excavated and dissected following the third harvest of aboveground biomass in November 2019. This was used to verify whether treatment effects observed in spring 2020 could be assigned to the presence of larvae and not to other factors. Roots excavated in autumn 2019 were randomly chosen from all blocks, treatments, and initial root size groups to provide a representative sub-sample. The remaining two thirds of roots were excavated in spring 2020 at the end of the experiment. All roots were placed in labeled plastic bags and stored in the dark at 4 °C until dissection, which took place right after the harvests in autumn 2019 and spring 2020.

Prior to dissection, roots were washed free of soil, weighed, and the number of primary and secondary roots and number of rosettes present at the root collar were counted. We used fresh weight of roots as the response variable because it allowed to relate final root mass directly to initial root mass in the analysis (see below) and to determine the 1:1 relation of steady-state of growth and decay (compare Fig. 4). Roots were then dissected to determine the probability of *R. obtusifolius* plants being infested by *Pyropteron* larvae. The presence of living and dead larvae was recorded, from which the total number of larvae was calculated (see Supplementary Appendix S1 for details on the determination of infestation probability). To receive a measure of herbivore load in relation to the final plant size of *R. obtusifolius*, the total number of larvae was divided by final root mass. Following dissection, the proportion of the root that was decayed (dead material, structure porous, color of material blackish, brownish) was visually estimated and assigned to one of six proportion categories (0, 0.05, 0.15, 0.50, 0.85, 1). We used categories because, after root dissection, a determination to precise proportions cannot be done anymore.

### Data analysis

Data were analyzed with (generalized) linear mixed-effects models. Response variables were infestation of *R. obtusifolius* plants by *Pyropteron* spp., total number of *Pyropteron* larvae retrieved per plant, total number of larvae per unit final root mass, aboveground biomass and number of rosettes per *R. obtusifolius* plant, root mass and number of roots per *R. obtusifolius* plant, and proportion of root decay. Predictor variables were harvest time (fixed factor with two levels), competition from *L. perenne* (fixed factor with two levels), *Pyropteron* treatments (fixed factor with three levels), and initial root mass. For initial root mass, we used the initially measured masses to increase accuracy and allow root mass to be a continuous variable. To account for the multilevel grouping structure of the experiment, block, main-plot, and sub-plot were each modeled as random factors (random intercepts). Given the different response variables and their underlying distributions, a variety of link functions were employed to appropriately model the data (see Supplementary Appendix S1 for all model equations). Note that a significant interaction between *L. perenne* competition and *Pyropteron* application in a generalised linear (mixed) model proves a synergism, i.e., an impact of the combined application of both factors being higher than the multiplicative effect of the two single factors.

Regarding predictors, the models had three different structures. First, we evaluated infestation of *R. obtusifolius* plants by *Pyropteron* ssp. in autumn 2019 and the following spring 2020. Thus, probability of infestation and total number of larvae per plant were modeled as a function of harvest time, *L. perenne* competition, *Pyropteron* treatments, and initial root mass (as defined above). Second, given that infestation with *Pyropteron* was confirmed and was similar in 2019 and 2020, all further response variables were analyzed for the final harvest in May 2020. Thus, aboveground biomass and final root mass of *R. obtusifolius*, number of larvae per unit final root mass, number of rosettes per *R. obtusifolius* plant, number of *R. obtusifolius* roots, and proportion of root decay each were regressed on *L. perenne* competition, *Pyropteron* treatments, and initial root mass. Third, visual inspection and preliminary tests indicated a significant competition × *Pyropteron* interaction on both aboveground biomass and final root mass of *R. obtusifolius* plants, meaning that the herbivore effect on *R. obtusifolius* would differ depending on *L. perenne* competition. Moreover, a second-order polynomial on initial root mass was needed to appropriately model the data. Therefore, after first testing for the main effects of *L. perenne* competition, *Pyropteron* treatment and their interaction, data for aboveground biomass and root mass was split into the *L. perenne* and no competition treatment to reduce model complexity and allow for a clearer interpretation. Here, predictors were *Pyropteron* treatments and a linear and quadratic term of initial root mass.

Final models included the two-way interactions of factor variables, while the inclusion of higher order interactions and interactions between factors and (continuous) initial root mass was assessed by the second-order Akaike Information Criterion (AICc^56^). Inference on main effects was achieved with single term deletion from the main effects model (each effect in turn) and subsequent likelihood ratio tests; interactions were similarly tested, but from a model that contained all interactions in that respective order. The marginal and conditional *R*^2^ of final models were calculated following Nakagawa and Schielzeth^57^ and Nakagawa et al.^58^. Differences in estimates between the factor levels of variables were tested post-hoc using the Tukey range test^59^. All data was analyzed with the statistical software R, version 4.2.2^60^, using the glmmTMB package for generalized linear mixed-effects models^61^ and the multcomp package for Tukey range tests^59^.

### Supplementary Information

Supplementary Information.

## Data Availability

The data generated and analyzed during the current study are available in the Zenodo data repository at 10.5281/zenodo.10044160.

## References

[1] Agrawal AA (2004). Resistance and susceptibility of milkweed: Competition, root herbivory, and plant genetic variation. Ecology.

[2] Cheng F, Cheng Z (2015). Research progress on the use of plant allelopathy in agriculture and the physiological and ecological mechanisms of allelopathy. Front. Plant Sci..

[3] Ferrero-Serrano Á, Collier TR, Hild AL, Mealor BA, Smith T (2008). Combined impacts of native grass competition and introduced weevil herbivory on Canada thistle (*Cirsium arvense*). Rangel. Ecol. Manag..

[4] Sheppard A, Smyth M, Swirepik A (2001). The impact of a root-crown weevil and pasture competition on the winter annual *Echium plantagineum*. J. Appl. Ecol..

[5] Sheppard AW, Moran VC, Hoffmann JH (1996). The interaction between natural enemies and interspecific plant competition in the control of invasive pasture weeds. Proceedings of the IX International Symposium on Biological Control of Weeds.

[6] Hambäck PA, Beckerman AP (2003). Herbivory and plant resource competition: A review of two interacting interactions. Oikos.

[7] Schwarzländer M, Hinz HL, Winston RL, Day MD (2018). Biological control of weeds: An analysis of introductions, rates of establishment and estimates of success, worldwide. BioControl.

[8] Agrawal AA, Van Zandt PA (2003). Ecological play in the coevolutionary theatre: Genetic and environmental determinants of attack by a specialist weevil on milkweed. J. Ecol..

[9] de Vries J, Evers JB, Dicke M, Poelman EH (2019). Ecological interactions shape the adaptive value of plant defence: Herbivore attack versus competition for light. Funct. Ecol..

[10] Friedli J, Bacher S (2001). Direct and indirect effects of a shoot-base boring weevil and plant competition on the performance of creeping thistle, *Cirsium*
*arvense*. Biol. Control.

[11] Kim TN, Underwood N, Inouye BD (2013). Insect herbivores change the outcome of plant competition through both inter- and intraspecific processes. Ecology.

[12] Morin L (2009). Review of approaches to evaluate the effectiveness of weed biological control agents. Biol. Control.

[13] Willis AJ, Berentson PR, Ash JE (2003). Impacts of a weed biocontrol agent on recovery from water stress in a target and a non-target *Hypericum* species. J. Appl. Ecol..

[14] Allen J (1974). Preliminary Observations and Investigations on Docks (Rumex spp) in Western Australia.

[15] Cavers PB, Harper JL (1964). *Rumex*
*obtusifolius* L. and *R*. *crispus* L. J. Ecol..

[16] Zaller JG (2004). Ecology and non-chemical control of *Rumex*
*crispus* and *R*. *obtusifolius* (Polygonaceae): A review. Weed Res..

[17] Pino J, Haggar RJ, Sans FX, Masalles RM, Hamilton RNS (1995). Clonal growth and fragment regeneration of *Rumex obtusifolius* L. Weed Res..

[18] Suter M (2023). Can the soil seed bank of *Rumex obtusifolius* in productive grasslands be explained by management and soil properties?. PLoS One.

[19] Grossrieder M, Keary IP (2004). The potential for the biological control of *Rumex obtusifolius* and *Rumex crispus* using insects in organic farming, with particular reference to Switzerland. Biocontrol News Inf..

[20] Niggli U, Nösberger J, Lehmann J (1993). Effects of nitrogen fertilization and cutting frequency on the competitive ability and the regrowth capacity of *Rumex*
*obtusifolius* L. in several grass swards. Weed Res..

[21] Finn JA (2013). Ecosystem function enhanced by combining four functional types of plant species in intensively managed grassland mixtures: A 3-year continental-scale field experiment. J. Appl. Ecol..

[22] Liebisch F (2013). Plant phosphorus nutrition indicators evaluated in agricultural grasslands managed at different intensities. Eur. J. Agron..

[23] Connolly J (2018). Weed suppression greatly increased by plant diversity in intensively managed grasslands: A continental-scale experiment. J. Appl. Ecol..

[24] Frankow-Lindberg BE (2012). Grassland plant species diversity decreases invasion by increasing resource use. Oecologia.

[25] Suter M, Hofer D, Lüscher A (2017). Weed suppression enhanced by increasing functional trait dispersion and resource capture in forage ley mixtures. Agric. Ecosyst. Environ..

[26] Hopkins A, Johnson RH (2002). Effect of different manuring and defoliation patterns on broad-leaved dock (*Rumex obtusifolius*) in grassland. Ann. Appl. Biol..

[27] Ringselle B (2019). Effects of renewal time, taproot cutting, ploughing practice, false seedbed and companion crop on docks (*Rumex* spp.) when renewing grassland. Eur. J. Agron..

[28] Scott JK, Sagliocco JL (1991). *Chamaesphecia doryliformis* [Lep, Sesiidae], a second root borer for the control of *Rumex s*pp [Polygonaceae] in Australia. Entomophaga.

[29] Scott JK, Sagliocco JL (1991). Host-specificity of a root borer, *Bembecia chrysidiformis* [Lep, Sesiidae], a potential control agent for *Rumex* spp [Polygonaceae] in Australia. Entomophaga.

[30] Fogliani, R. G. & Strickland, G. R. *Biological control of dock: Enhanced distribution of the dock moth*. Final report to Meat and Livestock Australia Project DAW.057 1994–2000 (Agriculture Western Australia, 2000).

[31] Hatcher P, Julien MH (2008). Biological control of *Rumex* species in Europe: Opportunities and constraints. Proceedings of the XII International Symposium on Biological Control of Weeds.

[32] Eilenberg J, Hajek A, Lomer C (2001). Suggestions for unifying the terminology in biological control. BioControl.

[33] Hahn MA, Schaffner U, Häfliger P, Lüscher A (2016). Establishment and early impact of the native biological control candidate *Pyropteron chrysidiforme* on the native weed *Rumex obtusifolius* in Europe. BioControl.

[34] Keary IP, Hatcher PE (2004). Combining competition from *Lolium perenne* and an insect-fungus combination to control *Rumex obtusifolius* seedlings. Weed Res..

[35] Jeangros B, Nösberger J (1990). Effects of an established sward of *Lolium*
*perenne* L. on the growth and development of *Rumex*
*obtusifolius* L. seedlings. Grass Forage Sci..

[36] Haag JJ, Coupe MD, Cahill JF (2004). Antagonistic interactions between competition and insect herbivory on plant growth. J. Ecol..

[37] Müller-Schärer H, Collins AR, Jorgensen SE (2012). Integrated weed management. Encyclopedia of Environmental Management.

[38] Müller-Schärer H (1991). The impact of root herbivory as a function of plant-density and competition: Survival, growth and fecundity of *Centaurea maculosa* in field plots. J. Appl. Ecol..

[39] Nyfeler D (2009). Strong mixture effects among four species in fertilized agricultural grassland led to persistent and consistent transgressive overyielding. J. Appl. Ecol..

[40] Rees M, Brown VK (1992). Interactions between invertebrate herbivores and plant competition. J. Ecol..

[41] Spafford H, Hawley J, Strickland G, Klinken RDV, Osten VA, Panetta FD, Scanlan JC (2008). Survival of dock moth larvae, *Pyropteron doryliformis* (Lepidoptera: Sesiidae), in tubers of fiddle dock (*Rumex pulcher*). Proceedings of the 16th Australian Weeds Conference.

[42] Fisher K, Fogliani R, Strickland G (1994). Biological Control of Dock: Field Augmentation.

[43] Klötzli J, Suter M, Lüscher A, Müller-Schärer H, Schaffner U (2022). Competitive interactions affect larval survival of two root-boring weed biological control candidates of *Rumex* spp. BioControl.

[44] Barbosa P (2009). Associational resistance and associational susceptibility: Having right or wrong neighbors. Annu. Rev. Ecol. Evol. Syst..

[45] Erb M, Lu J (2013). Soil abiotic factors influence interactions between belowground herbivores and plant roots. J. Exp. Bot..

[46] Shabbir A, Dhileepan K, Zalucki MP, Khan N, Adkins SW (2020). Reducing the fitness of an invasive weed, *Parthenium hysterophorus*: Complementing biological control with plant competition. J. Environ. Manage..

[47] Kim TN (2017). How plant neighborhood composition influences herbivory: Testing four mechanisms of associational resistance and susceptibility. PLoS One.

[48] Cipollini D (2004). Stretching the limits of plasticity: Can a plant defend against both competitors and herbivores?. Ecology.

[49] Santamaría J (2021). The role of competition and herbivory in biotic resistance against invaders: A synergistic effect. Ecology.

[50] Zhang Y, Meng H, Wang Y, He Q (2018). Herbivory enhances the resistance of mangrove forest to cordgrass invasion. Ecology.

[51] Bond W, Davies G, Turner R (2007). The Biology and Non-chemical Control of Broad-leaved Dock (Rumex obtusifolius L.) and Curled Dock (R. crispus L.).

[52] Pino J, Sans FX, Masalles RM (2002). Size-dependent reproductive pattern and short-term reproductive cost in *Rumex obtusifolius* L. Acta Oecol..

[53] Cottam D, Whittaker J, Malloch A (1986). The effects of chrysomelid beetle grazing and plant competition on the growth of *Rumex obtusifolius*. Oecologia.

[54] Schaffner U, Müller-Schärer H, Lüscher A, Kudsk P (2022). Integrated weed management in grasslands. Advances in Integrated Weed Management.

[55] Blossey B, Hunt-Joshi TR (2003). Belowground herbivory by insects: Influence on plants and aboveground herbivores. Annu. Rev. Entomol..

[56] Burnham KP, Anderson DR (2002). Model Selection and Multimodel Inference.

[57] Nakagawa S, Schielzeth H (2013). A general and simple method for obtaining *R*^2^ from generalized linear mixed-effects models. Methods Ecol. Evol..

[58] Nakagawa S, Johnson PCD, Schielzeth H (2017). The coefficient of determination *R*^2^ and intra-class correlation coefficient from generalized linear mixed-effects models revisited and expanded. J. R. Soc. Interface.

[59] Hothorn T, Bretz F, Westfall P (2008). Simultaneous inference in general parametric models. Biom. J..

[60] R Core Team. R: A language and environment for statistical computing. https://www.r-project.org/ (R Foundation for Statistical Computing, 2023).

[61] Brooks ME (2017). glmmTMB balances speed and flexibility among packages for zero-inflated generalized linear mixed modeling. R J..

[62] Garcia LC, Eubanks MD (2019). Overcompensation for insect herbivory: A review and meta-analysis of the evidence. Ecology.

[63] Agresti A, Coull BA (1998). Approximate is better than “exact” for interval estimation of binomial proportions. Am. Stat..

