# Supplementary material for: Synergistic effects of grass competition and insect herbivory on the weed Rumex obtusifolius in an inundative biocontrol approach
Source: Sci Rep. 2023 Oct 28;13:18508. doi: 10.1038/s41598-023-45609-y (PMC10613235; doi:10.1038/s41598-023-45609-y)
Supplement: Supplementary file 1 — Supplementary Information. [file 41598_2023_45609_MOESM1_ESM.pdf]

Supplementary Information

Synergistic effects of grass competition and insect herbivory on the weed *Rumex obtusifolius* in an inundative biocontrol approach

Julie Klötzli[1,2], Matthias Suter[1], Urs Schaffner[1,3], Heinz Müller-Schärer[2] & Andreas Lüscher[1]

[1]Agroscope, Forage Production and Grassland Systems, Reckenholzstrasse 191, 8046 Zurich, Switzerland

[2]Department of Biology/Ecology & Evolution, University of Fribourg, 1700 Fribourg, Switzerland

[3]CABI, Rue des Grillons 1, 2800 Delémont, Switzerland

Correspondence: Matthias Suter

Appendix S1 - Supporting text and information on the methods and analyses

Determination of infestation of *Rumex obtusifolius* with *Pyropteron* species

A plant was considered infested when at least one larva was retrieved. For roots harvested in spring 2020, a plant was also recorded as being infested when a head capsule or larval skin (both with head capsule width of more than 1300 µm), a silk chamber or a characteristic feeding tunnel with fresh frass was observed. Few larvae found in roots at spring 2020 were already in the pupa stage and were counted as alive. In spring 2020, the proportion of larvae alive was 0.88 and 0.68 for *P. chrysidiforme* and *P. doryliforme*, respectively.

Models to analyze the effects of harvest time, *Pyropteron* treatment, competition from *Lolium perenne*, and initial root mass on *Rumex obtusifolius* plant performance

This section complements the description of the (generalized) linear mixed-effects models in the main text. Only main effects models are presented. Model assumptions were evaluated by a simulation-based approach creating scaled residuals from the fitted model, using the R package DHARMa (Hartig, 2020).

Infestation probability

For infestation, only plants under the *Pyropteron* application treatments were analyzed because the control treatment (no inoculation with *Pyropteron*) was not infested with larvae. Doing so, the variable *Pyropteron* treatment had only two levels. Given that infestation was a binary response variable (1 if a plant was infested, 0 if not), the main effects model to estimate the probability of infestation was:

$$\text{Infestation} \sim Binomial(\pi)$$
$$E(\text{Infestation}) = \pi \qquad var(\text{Infestation}) = N\,\pi\,(1 - \pi)$$
$$\text{logit}(\pi) = \eta$$
$$\eta = \alpha + \beta\,\log(\text{Init\_Rm}) + \gamma\,\text{Harv} + \delta\,\text{Comp} + \kappa\,\text{Pdo} + \varphi_1\,\text{Block} + \varphi_2\,\text{Main-plot} + \varphi_3\,\text{Sub-plot} \qquad \text{(S1)}$$

where η denotes the survival probability on the link-scale in autumn 2019 at the no competition treatment (bare soil) and the application of *P. chrysidiforme.* With α being the intercept, the fixed β parameter estimates the effect of increasing initial root mass (Init_Rm), which – after

taking the natural logarithm – was centered around its mean to provide intuitive meaning to the estimates of factors (Schielzeth, 2010). The fixed γ parameter estimates the effect of the spring versus the autumn harvest (with Harv = 1 for the spring harvest, Harv = 0 otherwise); likewise, the fixed δ parameter estimates the effect of competition from *L. perenne* versus bare soil (with Comp = 1 for *L. perenne* swards, Comp = 0 otherwise). The fixed κ parameter estimates the effect of the *Pyropteron* treatment (with Pdo = 1 if the *Pyropteron* species was *R. doryliforme*, and Pdo = 0 otherwise). The random φ coefficients account for the block, main- and sub-plot effects. In particular, random coefficients φ_2 and φ_3 reflect the split-split plot structure of the design and capture potential correlation of response variables within main- and sub-plots. Yet, it turned out that the main-plot variance was generally estimated to be zero, and so it was omitted from all models.

Total number of larvae

Total number of larvae (Tot_Larv) per plant was a count variable with no overdispersion, the latter being approved by initial evaluation and likelihood ratio tests. Only infested plants were analyzed, meaning that the control treatment (no inoculation with *Pyropteron*) was omitted and the variable *Pyropteron* treatment had only two levels. The main effects model was:

$$\text{Tot\_Larv} \sim Poisson(\lambda)$$
$$E(\text{Tot\_Larv}) = \lambda \qquad var(\text{Tot\_Larv}) = \lambda$$
$$\log(\lambda) = \eta$$
$$\eta = \alpha + \beta \log(\text{Init\_Rm}) + \gamma \text{ Harv} + \delta \text{ Comp} + \kappa \text{ Pdo} + \varphi_1 \text{ Block} + \varphi_2 \text{ Sub-plot} \qquad \text{(S2)}$$

where η denotes the total number of larvae on the link-scale in autumn 2019 at the no competition treatment (bare soil) and the application of *P. chrysidiforme*, at mean log initial root mass. All remaining variables and parameters have meanings as explained.

Total number of larvae scaled by final root mass

The ratio of total number of larvae per 100 g of *final* root mass (Larv_Scal) was log transformed to perform a reasonable analysis (Jasienski & Bazzaz, 1999). Similar to total number of larvae, only infested plants were analyzed. The basic model was:

$$\text{Log(Larv\_Scal)} \sim Normal(\mu, \sigma^2)$$
$$E(\log(\text{Larv\_Scal})) = \mu \qquad var(\log(\text{Larv\_Scal})) = \sigma^2$$
$$\mu = \alpha + \beta \log(\text{Init\_Rm}) + \delta \text{ Comp} + \kappa \text{ Pdo} + \varphi \text{ Block} + \varepsilon \qquad \text{(S3)}$$

where μ is the estimated log ratio at the no competition treatment (bare soil) under the application of *P. chrysidiforme* at mean log initial root mass, and ε is assumed to be normally distributed with mean zero and variance σ^2. The random Sub-plot term had to be omitted due to convergence problems with the model fit. The inference for fixed effects was derived by single term deletion (each effect in turn) and subsequent *F*-tests for comparison of models. The approximate denominator degrees of freedom of terms were calculated using the Kenward–Roger method (Kenward & Rogers, 1997). Note that this regression analyzes the log ratio of two measured response variables, namely the number of larvae per plant and *final* root mass, and so Larv_Scal is not auto-correlated to the design factors including initial root mass.

Proportion of root decay

Proportion of root decay of *R. obtusifolius* plants was a continuous response variable in the standard unit interval, and was thus analyzed with beta regression (Ferrari & Cribari-Neto, 2004). To avoid the extremes 0 and 1, the variable (y) was first transformed to Decay_prop = $[y\,(N-1) + 0.5] \,/\, N$, with N being the sample size (Smithson & Verkuilen, 2006). Here, the control treatment was included and thus, the variable *Pyropteron* treatment had three levels. Using the parametrization of Smithson & Verkuilen (2006), the main effects model was:

$$\text{Decay\_prop} \sim Beta(a, b) \quad , \quad a = \theta\mu \quad , \quad b = \theta(1-\mu)$$

$$E(\text{Decay\_prop}) = \mu \qquad var(\text{Decay\_prop}) = \frac{\mu(1-\mu)}{(1+\theta)}$$

$$\text{logit}(\mu) = \eta$$

$$\eta = \alpha + \beta \log(\text{Init\_Rm}) + \delta\, \text{Comp} + \kappa_1\, \text{Pch} + \kappa_2\, \text{Pdo} + \varphi_1\, \text{Block} + \varphi_2\, \text{Sub-plot} \qquad \text{(S4)}$$

where η denotes the estimated proportion of root decay on the link-scale at the no competition treatment (bare soil) and controls (no *Pyropteron* species applied), at mean log initial root mass. The κ parameters estimate the effect of the *Pyropteron* treatment (with Pch = 1 if the applied *Pyropteron* species was *P. chrysidiforme*, and Pch = 0 otherwise; Pdo = 1 if the *Pyropteron* species was *R. doryliforme*, and Pdo = 0 otherwise).

Aboveground biomass and final root mass

Regarding aboveground biomass and final root mass of *R. obtusifolius*, the Gamma distribution was employed to provide strictly positive estimates and standard errors also for very low *R. obtusifolius* masses and to account for the strongly heteroscedastic variance over the range of initial root mass. Visual inspection of the data suggested strong effects of *L. perenne*

competition on both, aboveground biomass and final root mass, and the need to fit a second-order polynomial on initial root mass for a part of the data (compare Fig. 4, main text). Thus, we first fit a model to test for the main effects of *L. perenne* competition, *Pyropteron* treatment and their interaction, including initial root mass as a co-variate. For aboveground biomass (BM), the main effects model was:

$$\text{BM} \sim Gamma(\mu, \tau)$$

$$E(\text{BM}) = \mu \qquad var(\text{BM}) = \frac{\mu^2}{\tau}$$

$$\log(\mu) = \eta$$

$$\eta = \alpha + \beta_1 \log(\text{Init\_Rm}) + \beta_2 (\log(\text{Init\_Rm}))^2 + \delta\ \text{Comp} + \kappa_1\ \text{Pch} + \kappa_2\ \text{Pdo} + \varphi_1\ \text{Block} + \varphi_2\ \text{Sub-plot} \qquad \text{(S5)}$$

where η denotes BM at the no competition treatment (bare soil) and controls (no *Pyropteron* species applied), at mean log initial root mass. Moreover, when evaluated by likelihood ratio tests, it turned out that the variance of BM differed significantly over the range of initial root mass and between *L. perenne* competition and *Pyropteron* treatments. Therefore, in estimating parameters of equation (S5), τ was jointly modeled with:

$$\log(\tau) = \gamma_1 \log(\text{Init\_Rm}) + \gamma_2\ \text{Comp} + \gamma_3\ \text{Pch} + \gamma_4\ \text{Pdo} \qquad \text{(S6)}$$

An equivalent model was set up for final root mass of *R. obtusifolius*.

Results based on equation (S5) indicated a significant competition × *Pyropteron* interaction on both aboveground biomass and final root mass (see Table 2, main text), meaning that the herbivore effect on *R. obtusifolius* would differ depending on *L. perenne* competition. This justified to split the data into the *L. perenne* competition and the no competition treatment to reduce model complexity and allow for a clearer interpretation. Doing so, a second model tested for the *Pyropteron* treatment and its interaction with initial root mass (results in Supplementary Tables S1 and S2). Thus, for both aboveground biomass and final root mass, the linear predictor of the main effects model mass was:

$$\eta = \alpha + \beta_1 \log(\text{Init\_Rm}) + \beta_2 (\log(\text{Init\_Rm}))^2 + \kappa_1\ \text{Pch} + \kappa_2\ \text{Pdo} + \varphi_1\ \text{Block} + \varphi_2\ \text{Sub-plot} \qquad \text{(S7)}$$

where η denotes the response at the controls (no *Pyropteron* species applied) at mean log initial root mass. To consider the differing variance over the range of initial root mass and between *Pyropteron* treatments, the parameter τ was jointly modeled with:

$$\log(\tau) = \gamma_1 \log(\text{Init\_Rm}) + \gamma_2 \text{ Pch} + \gamma_3 \text{ Pdo} \quad \text{(S8)}$$

Number of rosettes

Total number of rosettes (No_Rosette) of *R. obtusifolius* plants was a count variable. Likelihood ratio tests on preliminary models revealed that the variable was overdispersed and therefore, to account for overdispersion, the *Negative Binomial* distribution was chosen to model the data. This led to:

$$\text{No\_Rosette} \sim \textit{Negative Binomial}(\mu, k)$$

$$E(\text{No\_ Rosette}) = \mu \qquad var(\text{No\_ Rosette}) = \mu + \frac{\mu^2}{k}$$

$$\log(\mu) = \eta$$

$$\eta = \alpha + \beta \log(\text{Init\_Rm}) + \delta \text{ Comp} + \kappa_1 \text{ Pch} + \kappa_2 \text{ Pdo} + \varphi_1 \text{ Block} + \varphi_2 \text{ Sub-plot} \quad \text{(S9)}$$

where η denotes the number of rosettes on the link-scale at the no competition treatment (bare soil) and controls (no *Pyropteron* species applied), at mean log initial root mass. The dispersion parameter k revealed significant dependence on *Pyropteron* treatment, and so it was modeled with:

$$\log(k) = \gamma_1 \text{ Pch} + \gamma_2 \text{ Pdo} \quad \text{(S10)}$$

Number of roots

Finally, total number of roots (No_Roots) of *R. obtusifolius* plants was a count variable with substantial overdispersion. The main effects model was thus equivalent to equation (S10). No extra dispersion model was needed to fit this data.

References

Ferrari, S.L.P. & Cribari-Neto, F. (2004) Beta regression for modelling rates and proportions. *Journal of Applied Statistics* **31**, 799–815.

Hartig, F. (2022) DHARMa: Residual diagnostics for hierarchical (multi-level/mixed) regression models. R package version 0.4.6. R Foundation for Statistical Computing, Vienna, Austria. https://cran.r-project.org/web/packages/DHARMa/

Jasienski, M. & Bazzaz, F.A. (1999) The fallacy of ratios and the testability of models in biology. *Oikos* **84**, 321–326.

Kenward, M.G. & Roger, J.H. (1997) Small sample inference for fixed effects from restricted maximum likelihood. *Biometrics* **53**, 983–997.

Schielzeth, H. (2010) Simple means to improve the interpretability of regression coefficients. *Methods in Ecology and Evolution* **1**, 103–113.

Smithson, M. & Verkuilen, J. (2006) A better lemon squeezer? Maximum-likelihood regression with beta-distributed dependent variables. *Psychological Methods* **11**, 54–71.

Appendix S2 - Supplementary Tables and Figures

Table S1. Summary of generalized linear mixed-effects models for the effects of *Pyropteron* application treatment and initial root mass of *R. obtusifolius* on its aboveground biomass and final root mass in spring 2020. Data was split into the no competition and *L. perenne* competition treatment to reduce model complexity. See Table S2 for the corresponding regression coefficients under *L. perenne* competition.

		Aboveground biomass[#]				Final root mass			
		No competition		*L. perenne* competition		No competition		*L. perenne* competition	
Variable	df	χ^2	*P*	χ^2	*P*	χ^2	*P*	χ^2	*P*
Log(Initial root mass), (linear)[§]	1	11.5	< 0.001	50.8	< 0.001	18.7	< 0.001	84.8	< 0.001
Log(Initial root mass)^2, (quadratic)[§]	1	0.1	0.738	7.5	0.006	5.0	0.025	3.6	0.059
Pyropteron treatment	2	0.8	0.655	9.9	0.007	0.9	0.635	10.2	0.006
Log(Initial root mass) × *Pyropteron* treatment	2	–[‡]	–[‡]	5.4	0.066	–[‡]	–[‡]	4.6	0.101
R^2_m		0.097		0.607		0.231		0.738	
R^2_c		0.156		0.696		0.290		0.745	

R^2_m and R^2_c: marginal and conditional R^2. Low R^2 values under no competition are due to a flat response (compare Fig. 4a,c, main text)

[#]Cumulative biomass over five harvests

[§]Total effects across all *Pyropteron* treatments (incl. controls) based on single term deletion from the main effects model

[‡]Term not included, based on evaluation with the AICc

Table S2. Regression coefficients (on link-scale) from generalized linear mixed-effects models for the effects of *Pyropteron* application treatment and initial root mass of *R. obtusifolius* on its aboveground biomass and final root mass *under competition from a L. perenne* sward in spring 2020. Effects of the *P. chrysidiforme* application treatment are in **bold**.

	Aboveground biomass[#]			Final root mass		
Variable	Estimate (SE)	z-value	*P*	Estimate (SE)	z-value	*P*
Intercept (Control)[§]	0.457 (0.242)	1.89	0.059	3.188 (0.144)	22.08	< 0.001
Log(Initial root mass), (linear)[‡]	0.869 (0.111)	7.81	< 0.001	0.770 (0.093)	8.32	< 0.001
Log(Initial root mass)^2, (quadratic)[‡]	-0.157 (0.059)	-2.99	0.003	-0.073 (0.036)	-2.03	0.042
P. chrysidiforme	-0.875 (0.222)	**-3.94**	**< 0.001**	-0.537 (0.154)	**-3.48**	**< 0.001**
P. doryliforme	-0.409 (0.254)	-1.61	0.107	-0.405 (0.164)	-2.47	0.013
**Log(Initial root mass) × *P. chrysidiforme***[¶]	0.446 (0.195)	**2.29**	**0.022**	0.227 (0.119)	**1.90**	**0.057**
Log(Initial root mass) × *P. doryliforme*[¶]	0.009 (0.231)	0.04	0.969	0.019 (0.120)	0.16	0.877

[#]Cumulative biomass over five harvests

[§]Control: no *Pyropteron* species applied

[‡]Slope estimates for control treatment

[¶]Difference in slopes to the control treatment under *P. chrysidiforme* and *P. doryliforme* application, respectively

Table S3. Summary of generalized linear mixed-effects models for the effects of competition from a *L. perenne* sward, *Pyropteron* application treatment, and initial root mass of *R. obtusifolius* on its total number of roots and number of rosettes of in spring 2020.

		Number of rosettes		Total number of roots	
Variable	df	χ^2	P	χ^2	P
Log(Initial root mass)	1	45.1	< 0.001	70.2	< 0.001
Competition	1	112.0	< 0.001	94.9	< 0.001
Pyropteron treatment	2	4.0	0.136	1.3	0.512
Competition × *Pyropteron* treatment	2	0.8	0.669	1.3	0.511
Log(Initial root mass) × Competition	1	13.3	< 0.001	9.0	0.003
R^2_m		0.761		0.673	
R^2_c		0.778		0.703	

R^2_m and R^2_c: marginal and conditional R^2, respectively

Nakagawa, S. & Schielzeth, H. (2013) A general and simple method for obtaining R^2 from generalized linear mixed-effects models. *Methods in Ecology and Evolution* **4**, 133–142.

Nakagawa, S., Johnson, P.C.D. & Schielzeth, H. (2017) The coefficient of determination R^2 and intra-class correlation coefficient from generalized linear mixed-effects models revisited and expanded. *Journal of the Royal Society Interface* **14**, 20170213.

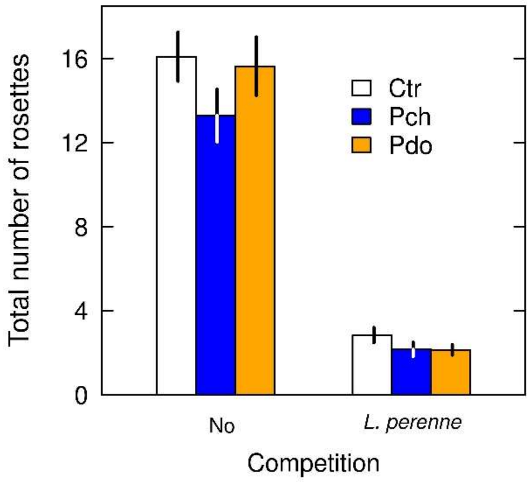

Figure S1. Total number of rosettes in spring 2020 as affected by *Pyropteron* treatment (no application [Ctr], application of *P. chrysidiforme* [Pch] or *P. doryliforme* [Pdo]) under no competition and competition from a *L. perenne* sward. Displayed are means ± standard error across all initial root sizes.

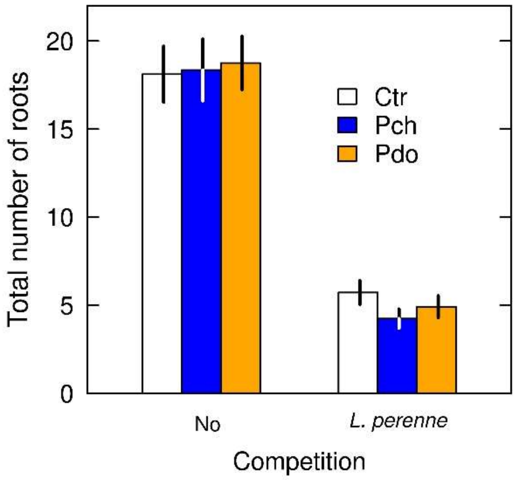

Figure S2. Total number of roots in spring 2020 as affected by *Pyropteron* treatment (no application [Ctr], application of *P. chrysidiforme* [Pch] or *P. doryliforme* [Pdo]) under no competition and competition from a *L. perenne* sward. Displayed are means ± standard error across all initial root sizes.
